# Development of the SPUR tool: a profiling instrument for patient treatment behavior

**DOI:** 10.1186/s41687-022-00470-x

**Published:** 2022-06-06

**Authors:** Béatrice Tugaut, Selam Shah, Kevin Dolgin, Hanna Rebibo Seror, Benoit Arnould, Marie-Eve Laporte, Aaron Lee, Lydiane Nabec, Reem Kayyali, Joshua Wells, John D. Piette, Guillaume Hubert

**Affiliations:** 1 Patient Centered Outcomes, ICON plc, 27 rue de la Villette, 69003 Lyon, France; 2 Patient Centered Outcomes, ICON plc, Boston, MA USA; 3 Observia, Paris, France; 4 IAE Paris 1 Panthéon-Sorbonne (Sorbonne Business School), Paris, France; 5 Department of Psychology, University of Mississippi, Oxford, MS USA; 6 Université Paris-Saclay, Paris, France; 7 Pharmacy Department, Kingston University, Kingston Upon Thames, UK; 8 Department of Health Behavior and Health Education, University of Michigan School of Public Health, Ann Arbor, MI USA

**Keywords:** Literature review, Interview, Cognitive test, Translation, Compliance, Health beliefs

## Abstract

**Background:**

Long-term treatment adherence is a worldwide concern, with nonadherence resulting from a complex interplay of behaviors and health beliefs. Determining an individual’s risk of nonadherence and identifying the drivers of that risk are crucial for the development of successful interventions for improving adherence. Here, we describe the development of a new tool assessing a comprehensive set of characteristics predictive of patients’ treatment adherence based on the Social, Psychological, Usage and Rational (SPUR) adherence framework. Concepts from existing self-reporting tools of adherence-related behaviors were identified following a targeted MEDLINE literature review and a subset of these concepts were then selected for inclusion in the new tool. SPUR tool items, simultaneously generated in US English and in French, were tested iteratively through two rounds of cognitive interviews with US and French patients taking long-term treatments for chronic diseases. The pilot SPUR tool, resulting from the qualitative analysis of patients’ responses, was then adapted to other cultural settings (China and the UK) and subjected to further rounds of cognitive testing.

**Results:**

The literature review identified 27 relevant instruments, from which 49 concepts were included in the SPUR tool (Social: 6, Psychological: 13, Usage: 11, Rational: 19). Feedback from US and French patients suffering from diabetes, multiple sclerosis, or breast cancer (n = 14 for the first round; n = 16 for the second round) indicated that the SPUR tool was well accepted and consistently understood. Minor modifications were implemented, resulting in the retention of 45 items (Social: 5, Psychological: 14, Usage: 10, Rational: 16). Results from the cognitive interviews conducted in China (15 patients per round suffering from diabetes, breast cancer or chronic obstructive pulmonary disease) and the UK (15 patients suffering from diabetes) confirmed the validity of the tool content, with no notable differences being identified across countries or chronic conditions.

**Conclusion:**

Our qualitative analyses indicated that the pilot SPUR tool is a promising model that may help clinicians and health systems to predict patient treatment behavior. Further steps using quantitative methods are needed to confirm its predictive validity and other psychometric properties.

**Supplementary Information:**

The online version contains supplementary material available at 10.1186/s41687-022-00470-x.

## Background

In its 2003 call to action, the World Health Organization stated that “increasing the effectiveness of adherence interventions may have a far greater impact on the health of the population than any improvement in specific medical treatments” while pointing out that average adherence rates for chronic treatments hover at around 50% [1]. In 2018, the OECD stated that “Despite mounting evidence, amassed for more than four decades, poor adherence to medications still affects approximately half of the population that receives prescriptions, leading to severe health complications, premature deaths, and an increased use of healthcare services” [2].

Adherence behaviors are known to result from a complex interplay of health beliefs and other factors that can make it difficult to identify the causes of nonadherence or the most successful strategies for addressing the problem in a given treatment plan [3–5]. In order to create support programs that can improve adherence, the determinants of initial prescription filling, prescription refilling, and discontinuation, must be understood. To make this knowledge useful clinically, a holistic patient profiling tool is needed to easily determine both an individual’s risk of nonadherence and the drivers of that risk.

Several conceptual models have been developed to describe the patient-level drivers of medication adherence. These include the Behavior Change Wheel [6], the Theory of Planned Behavior (TPB), and the Health Belief Model (HBM) [7, 8]. While useful, these models have their limitations. For example, the applicability of the TPB in cases of curative behaviors such as adherence to medication for chronic disease has been called into question [9, 10]. Moreover, the HBM does not address the noncognitive components of patient behavior, such as affective factors, medication costs and psychological traits like reactance [10]. Patients’ beliefs and relationships with their health system and treatment team can have a lasting impact on the way patients perceive a treatment, and may contribute to an additional emotional and psychological burden, especially when the treatment has serious side effects [11].

A number of measures exist that are predictive of adherence behavior, but they focus on the risk of nonadherence and typically offer no associated measures to examine the drivers of this risk. In order to provide clinicians and health systems with a reliable, comprehensive, but practical tool for measuring the drivers of treatment nonadherence, we developed a multidimensional questionnaire, taking into account the features of the main theoretical frameworks in the literature, while maintaining a level of simplicity allowing providers to easily administer an assessment based on patient-reported information. This tool was developed to provide a measure of risk of nonadherence while grouping the drivers of adherence into four categories and providing measures for each of these categories [12]:

1. *Social:* Social factors include both the impact of those close to the patient and the role of society as a whole (e.g., the influence of sociocultural expectations).
2. *Psychological:* Psychological factors include three nonclinical psychological factors: self-concept (tendency to deny the existence or extent of the illness), reactance (tendency to resist authority) and the discounting of future values (degree to which asymmetrical valuing of short-term effects over long-term effects affects behavior).
3. *Usage:* Usage factors correspond to control elements (such as perceived self-efficacy) and to practical concerns with respect to self-medication and access to medication (nonaccess to medication due to physical constraints; financial difficulty; difficulty with self-administration).
4. *Rational:* Rational factors focus on perceptions of the treatment’s benefits, threats, the seriousness of the illness, and the individual’s risk for bad outcomes.

This framework is abbreviated by the acronym SPUR, standing for Social, Psychological, Usage and Rational. We propose that a profiling tool can measure these different elements for a given patient, and we hypothesize that its results will be predictive of adherence behavior. Furthermore, by providing not only a measure of behavioral risk but a set of explicit measures of the drivers of risk, such a tool can help in the customization of support efforts for each patient, whether in a digital support program or in a guided interpersonal setting.

We developed the SPUR tool so that clinicians and health systems can use it to assess patients’ characteristics predictive of their treatment adherence and understand the drivers of that risk. The tool is based on a review of published literature, input from behavioral scientists and clinicians, and feedback from patients living with a range of chronic diseases. The SPUR tool is designed to be applicable to adult patients prescribed with long-term treatment for a chronic condition and without major functional limitations such as dementia that prevent them from completing the SPUR evaluation independently. The purpose of the current paper is to describe the development of the SPUR tool including the initial evidence-based literature review used to define the factors and identify items, and the process of initial cognitive testing.

## Methods

### International Advisory Board

**Fig. 1:**
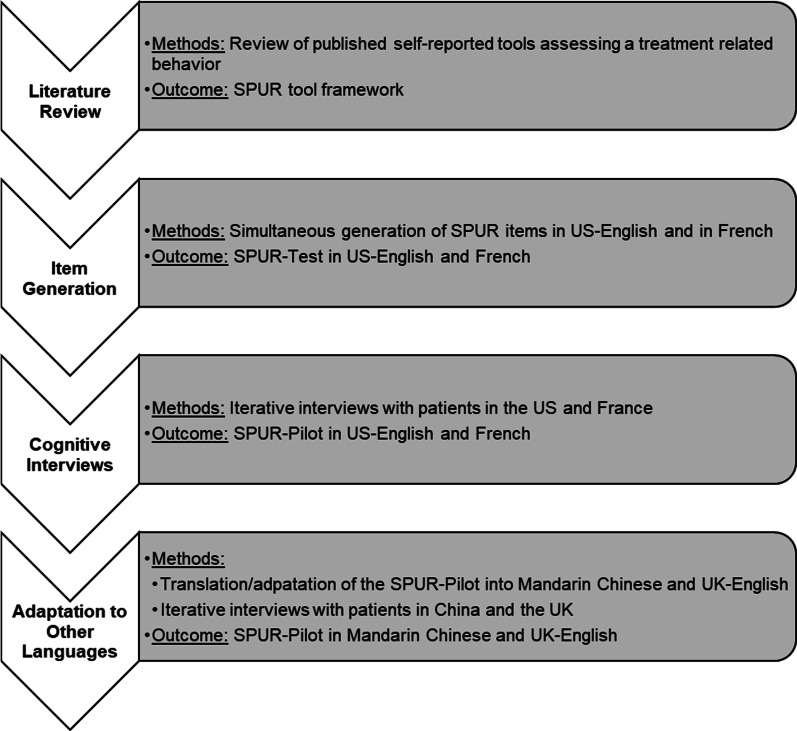
Development steps of the SPUR tool. *SPUR* Social, Psychological, Usage, Rationale

An international multidisciplinary group of experts in chronic disease management, medication adherence, and the development of patient-reported outcome measures was recruited to provide clinical input and assess scientific relevance throughout the qualitative steps of developing the SPUR tool (Fig. 1). The International Advisory Board (IAB) served in a consultative role, giving guidance to a working group (including BT, SS, BA, KD and GH) who developed the conceptual framework of the SPUR tool and drafted its items. The IAB consisted of eight members (JP, AL, RK, LN, MEL, BA, KD, and GH) from three countries (USA, UK, and France) who are experts in chronic disease management (JP, AL), the optimization of public health and care of patients with long term conditions (RK), consumer behavior (LN and MEL), PROs (BA), and behavioral science and the application of digital tools to patient support programs (KD and GH). The IAB met via teleconference on a quarterly basis, but meetings were conducted more often when new data became available during the testing process. The meetings typically lasted 2 h.

### Review of published self-reported tools related to adherence behaviors

A targeted literature search was performed to identify and critically review existing tools assessing treatment-related behaviors, as well as published theories and frameworks for the determinants of adherence. Based on this review, the initial SPUR framework was constructed. The literature search focused on publications in English indexed in MEDLINE from 2013 to 2018. The search strategy is detailed in Table 1. References were selected if they included a description of the development and/or the validation of a self-report tool assessing a treatment-related behavior or if they included explicit reference to at least one behavioral framework. Additional references were provided by the IAB. Abstracts and articles were rejected if they were out of scope, had no mention of a tool, or were not sufficiently detailed. Tools mentioned in the identified set of articles were reviewed in detail. Key articles were abstracted to identify information including the study’s objectives, target population, number of items, domains, references, and availability of a review copy of the described tool. Items and their underlying detailed concepts were screened individually. Items were rejected if (1) they were designed specifically for a single disease or a treatment, (2) they were found in multiple tools (in that case, the item was kept only once), (3) they assessed the number of doses missed and, (4) they were worded as an open-ended question. The concepts related to selected items were then categorized according to the four dimensions of the SPUR framework (i.e., Social, Psychological, Usage, or Rational), and then similar concepts were combined. Item selection, concept categorization and merging were reviewed and discussed iteratively with the IAB to ensure that each item was attributed to the relevant SPUR dimension and that each SPUR dimension was well addressed by the group of retained items.

**Table 1:** Search strategy

Terms/parameters	Keywords	Commands
1.	(Health Belief Model* OR HBM or Theory of Planned Behav* OR TPB OR Transtheoretical model* OR TTM OR (capability AND opportunity AND motivation) OR COM-B OR COMB OR illness perception* OR (belie* AND medicine*) OR beliefs about medicines questionnaire OR BMQ) [Title or abstract]	AND
2.	(patient-reported outcome* OR pro OR questionnaire* OR instrument* OR scale* OR tool* OR survey*) [Title or abstract] OR (Surveys and Questionnaires) [MeSH Term]	AND
3.	(complian* OR adheren* OR persisten* OR noncomplian* OR nonadheren* OR nonpersisten* OR non complian* OR non adheren* OR non persisten*) [Title or abstract]	AND
Limits	Species: Humans	
	Languages: English	
	Years: Past 5 years	

*Unlimited truncation; any word beginning with the term is searched

### Item generation

From the list of concepts issued from the literature review, new items were generated simultaneously in French and US English by native speakers of each target language. For each concept, at least one item was generated, and each new item was compared across the two languages to ensure conceptual equivalence and relevance. The first test version of the US English and French SPUR tool (SPUR-Test-1) was reviewed and approved by the IAB before the cognitive interviews.

### Cognitive interviews

The cognitive interviews aimed to incorporate a diverse sample of patients living with chronic diseases to provide feedback on (1) the relevance of the concepts covered by the tool; and (2) the ease of comprehension, cross-cultural validity, clarity, appropriateness of wording, and acceptability of the tool.

Two rounds of cognitive interviews were performed with patients taking long-term treatments for chronic diseases [13]. A total sample of 30 patients were enlisted through recruiting agencies in the US (n = 15) and through Observia’s network in France (n = 15). To be eligible, adult patients had to self-report either type 2 diabetes (T2D), multiple sclerosis (MS) or breast cancer (BCA) using hormone therapy. Local native speakers used a structured patient interview guide containing SPUR-Test-1 (Additional file 1) to conduct the 1-h phone interviews in each country. Briefly, patients were first asked for their general impression of the questionnaire (comprehension, layout, length, etc.) and their opinion on the title, instructions, and scale. For each of the items, patients were asked their opinion about the relevance of the item to their treatment behavior and the ease of comprehension. Comprehension was tested using a teach-back method, in which the patient was asked to restate the item in their own words. Suggestions for additional concepts or items were probed. Sociodemographic characteristics were also collected at the end of the interview. With the patient’s consent, each interview was audio-recorded to facilitate the discussion and the qualitative analysis. The analytical approach to cognitive interviews was straightforward and pragmatic: responses related to each of the elements of the questionnaire that were evaluated (i.e., instructions, scales, relevance, item wording, etc.) were noted verbatim in a table for each patient. The main themes of the responses to each of the elements were then collated and reviewed by the IAB. Suggested changes were then discussed and any changes made were agreed by all IAB members before being implicated. The results of the qualitative analysis were used to adapt and improve the SPUR tool following an iterative process. Modifications resulting from the initial round of interviews (items deleted, added, reformulated, or kept as they were) resulted in the SPUR-Test-2 version. A second round of cognitive interviews was then conducted by the same interviewers with a new set of patients using the same interview guide containing the SPUR-Test-2 tool instead of SPUR-Test-1. Final item wording was determined, and consistency of item understanding across the French and US English versions was independently evaluated and compared by local native speakers. This resulted in the pilot version of the SPUR tool (SPUR-Pilot).

### Adaptation to Mandarin and UK English

The SPUR-Pilot was then translated from US English into Mandarin for a follow-up study in China. Special care was taken during translation of the concept of “treatment” to differentiate Western treatment from traditional Chinese medicine. To ensure cross-cultural validity, cognitive interviews were carried out with Chinese patients following the same methodology as that used in France and in the US. The follow-up study included two rounds of cognitive interviews with a total of 30 Chinese patients with T2D, BCA, or chronic obstructive pulmonary disease (COPD). Chinese patients were recruited via a recruiting agency and interviewed via telephone by a native Mandarin speaker.

The US English version of the SPUR-Pilot was also adapted into a UK English version. Members of the IAB (RK and JW) were involved in the adaptation of the SPUR tool to UK English. The SPUR-Pilot was submitted by telephone to 15 patients with a chronic disease recruited via community pharmacists in the UK for one round of cognitive interviews.

All patients received a financial incentive for giving their time to participate in the interviews.

## Results

### Development of the SPUR tool

**Fig. 2:**
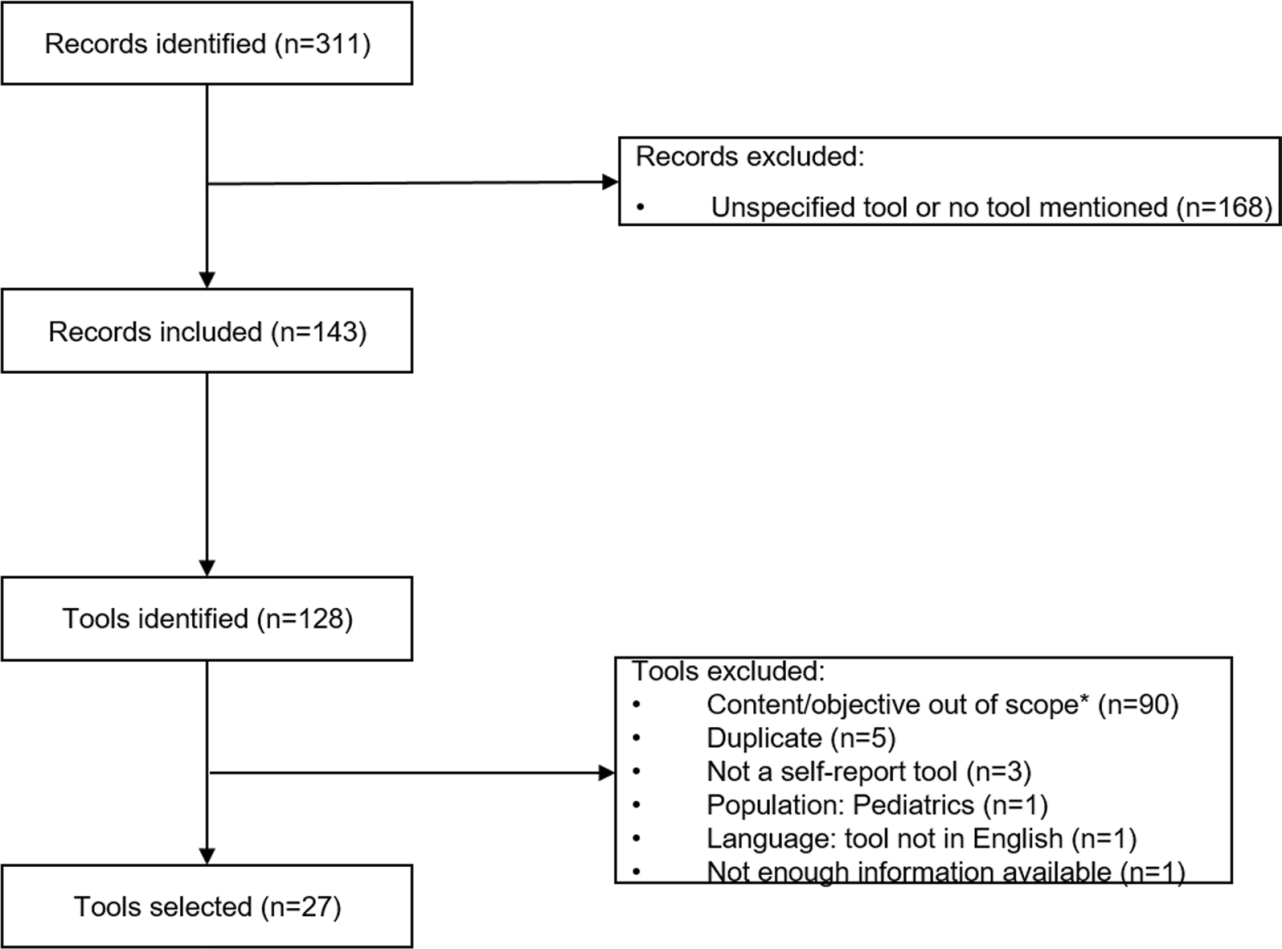
Selection process of the targeted literature review. *Not related to any of the concepts of interest (i.e., treatment and treatment adherence)

The literature search retrieved 311 abstracts: 143 references were included for further consideration and 128 unique tools were identified (see Fig. 2). Twenty-seven tools that reflected the published theories and frameworks used to frame the SPUR tool were identified for further review. A brief description of the tools we included is presented as Additional material (Additional file 2). From the 27 tools, 621 items were selected. Items were excluded because they were open-ended questions (n = 10), described a medication regimen (n = 3), concerned missed doses (n = 15), were disease specific (n = 94), or because of other reasons mainly related to clarity of concept (n = 25). The elimination of duplicates resulted in the exclusion of another 57 items. The remaining 417 items were categorized into 49 detailed concepts as the framework of the SPUR tool.

SPUR-Test-1 contained 49 items (one new item developed for each identified detailed concept) organized into four groups of factors according to the SPUR framework: Social (6 items), Psychological (13 items), Usage (11 items), Rational (19 items). Items were worded as statements describing disease- and treatment-related behaviors. Instructions were to choose the best response using a 10-point numerical rating scale (NRS) from 1 = “I strongly disagree” to 10 = “I strongly agree”. The wording of the items was generic so that they could be adapted to a target condition (e.g., “My [health problem] affects my social life” could be adapted to “My diabetes affects my social life”).

### Cognitive interviews

In total, 30 patients were interviewed (n = 14 for the first round; n = 16 for the second round) in the initial phase, including patients in France (n = 7 in the first round and n = 8 in the second round) and in the US (n = 7 in the first round and 8 in the second round). Patients’ sociodemographic and clinical characteristics are presented in Table 2. Participants were between 35 and 77 years of age (median: 51 and 58 for the American and French patients, respectively). Self-reported conditions included T2D (n = 9), MS (n = 11) and BCA (n = 10).

**Table 2:** Sociodemographic and clinical data of patients from France, US, China and UK

	US (n = 15)	France (n = 15)	China (n = 30)	UK (n = 15)
Condition (n)				
Type 2 diabetes	5	4	10	15
COPD	0	0	10	0
Multiple sclerosis	5	6	0	0
Breast cancer with hormone therapy	5	5	10	0
Gender (n)				
Male/female	5/10	4/11	15/15	5/10
Age (years)				
Median (min–max)	51 (35–71)	58 (38–77)	54 (32–63)	55 (30–80)
Educational level (n)				
Some high school	0	1	1	2
High school	4	3	13	3
Bachelor’s degree	2	3	12	6
Post-graduate degree	6	8	3	4
Other	3	0	0	0
Employment status (n)				
Full-time	2	5	7	5
Part-time	3	1	7	1
Retired	2	7	13	8
Unemployed	7	2	3	1

*COPD* chronic obstructive pulmonary disease

For the first round of cognitive interviews, patients in both countries reported that, overall, the tool was clear, easy to understand and comprehensive (“it was very easy to read and understand the questionnaire”). Instructions were reported to be clear and understandable (“self-explanatory”, “brief and to the point”, “clear instructions”). Patients found the tool’s completion time short (even though they took approximatively 20 min to complete it: “short questionnaire, filled out fairly quickly”, “same time as usual questionnaires”), and the layout was well accepted (“format is very clear”, “the format/layout is great”, “very well done”, “I like the dark font”). For the most part, patients found the topics of high interest and relevant to their daily experience with their specific treatment (“it asked good questions about my condition [diabetes] and made me think”, “Questions were really good in terms of how people react to their diagnosis/disease and how it affects the relationships in their life”). In general, feedback was consistent across patients with the same chronic condition. Patients gave some suggestions for improving the tool. For example, the 10-point NRS scale was often reported to be too fine-grained with too many choices: “Difficult to differentiate between response modalities, between numbers (e.g., 6 vs. 7)”, “Too many response choices”, “There are too many responses, too much subtlety”, “The scale was too long”, “Put fewer answers, e.g., 1–6”. Patients also identified issues including redundancy across items, desirability effects, and some invariant responses in some of the items. Country/language-specific issues were also identified. For example, patients in the US, but not France, identified the importance of medication costs as a driver of their adherence. Detailed results of the two rounds of cognitive interviews of the US English version of the whole questionnaire are presented in Additional file 3.

**Fig. 3:**
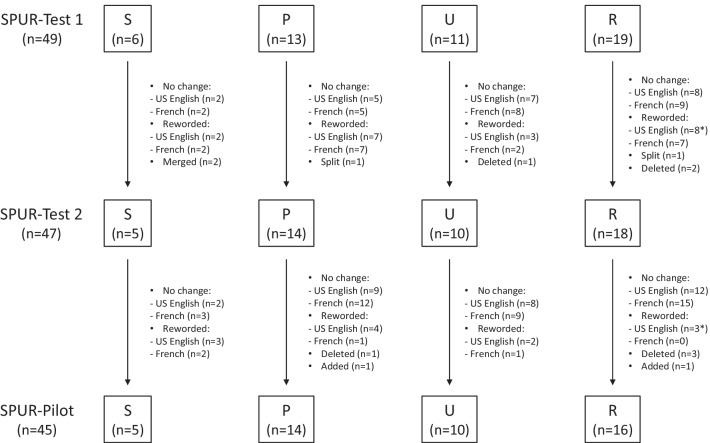
Evolution of the SPUR tool versions (US English and French). *SPUR* Social, Psychological, Usage, Rationale, *n* number of items. *Example of rewording (US English version): SPUR-Test 1: “Medications should only be taken when needed”; SPUR-Test 2: “I believe I can stop taking my treatment for my [health problem] when I feel better.”; SPUR-Pilot: “I believe I can stop my treatment for my [health problem] when I feel better”

Following the first round of cognitive interviews, two items were merged, three items were deleted, two items were divided into two items, and the others were either kept as they were (n = 22 in US English; n = 24 in French) or reworded (n = 20 in US English; n = 18 in French) (Fig. 3 and Additional file 3). The modified version (SPUR-Test-2) contained 47 items organized into the same four sections: Social (5 items), Psychological (14 items), Usage (10 items), Rational (18 items). The NRS was shortened to 5 points.

Based on these modifications, interviews with new patients in the second round confirmed that the tool was clear and easy to understand (“pretty straightforward and simple”, “very straightforward without using a bunch of words”), and an improved acceptability of the short 5-point NRS was reported: “5-point Likert is good; 3 is for "I don’t know" or "Yes and No"”, “Five boxes is good, it gives enough choices”. After the second round of cognitive interviews, four items were deleted, two items were added, and others were slightly reworded (n = 12 for the US English version; n = 4 for the French version) (Fig. 3 and Additional file 3). It was also noted that in future interviews there was a need to specify whether the items concerned a specific treatment or all treatments being taken by the patient as some polymedicated patients with several comorbidities found it difficult to answer questions regarding their medication adherence.

The resulting SPUR-Pilot tool (Additional file 4) to be used for future psychometric testing contained 45 items: Social (5 items), Psychological (14 items), Usage (10 items), Rational (16 items).

### Adaptation to Mandarin and UK English

In total, 30 patients aged between 32 and 63 years (median: 54) were interviewed in China (n = 15 per round). The chronic conditions self-reported by these patients included T2D (n = 10), BCA (n = 10), and COPD (n = 10) (Table 2). The SPUR-Pilot was well accepted and well understood by the Chinese patients: “The questions are well designed, though repeated in the meaning somehow, it seems the designer tries to cross check the answer from different angles”, “All the questions are easy to understand”, “Not difficult, I can easily understand”, “I like the design and the order of the questionnaire, it is like chatting with an old friend”. Following the first round of cognitive interviews, no major changes were made because the tool was deemed understandable and culturally acceptable. Three items were considered repetitive and ten items were slightly reworded for clarity. These modifications did not impact the wording and the structure of the original US and French versions. No changes were performed after the second round of interviews.

In the UK, 15 patients with self-reported T2D aged between 30 and 80 years (median: 55) were interviewed in one round (Table 2). UK patients felt that the items were easy to understand and were relevant to their condition. No subsequent changes were made to the UK English version after the cognitive interview.

## Discussion

Our main aim in creating the SPUR assessment tool was to develop an instrument that would allow clinicians and health systems to comprehensively identify patient-level drivers of medication adherence in the context of chronic disease management. Based on a targeted review of the literature, input from an international panel of experts, and a multinational effort to engage patients in revising the items, we developed a 45-item tool in accordance with the SPUR adherence framework. The qualitative assessments conducted during the initial phase helped to optimize the SPUR tool and ensure that the items included were relevant to a diverse group of patients with several different chronic diseases and allowed for a comprehensive assessment of main drivers of medication adherence, while making sure that the tool remained straight-forward to use and easy to understand. The patient responses were generally homogeneous across France and the US, and the follow-up qualitative study in China and the UK showed that the tool could be successfully adapted for use across cultures.

Patient feedback indicated that the items included in the tool, which were generated from a list of concepts relevant to the SPUR conceptual framework identified through a targeted literature review, were highly relevant for the daily challenges faced by patients affected by a chronic disease in adhering to their treatment. In addition, special care was taken when items were generated so that the SPUR tool could be used with patients who had been diagnosed with a variety of common chronic diseases. Indeed, as each medication and disease has its own unique characteristics, we ensured that the patients selected for cognitive interviews in our study presented different chronic conditions, including T2D, MS and BCA. Moreover, to ensure that the tool was suitable for use in a wide range of patient contexts, we enrolled a diverse group of patients (in terms of gender, age, employment status, and cultural and educational background) to provide input on the tool development. The patient responses obtained in our study confirmed that the wording of the items and underlying concepts made sense to this diverse range of patients, regardless of their chronic disease.

The iterative tests also allowed the response option format of the SPUR tool to be assessed and improved. Adjustments made during the first round of cognitive interviews allowed the cognitive effort needed to complete the assessment to be reduced: switching from a 10-point NRS scale to using the same 5-point NRS scale throughout enhanced the simplicity of the tool. All the patients interviewed after this switch were satisfied with the 5-point NRS scale, regardless of their chronic disease.

The qualitative assessments during the development of the SPUR tool provided encouraging early findings in terms of the cross-cultural validity of the tool. This was of particular importance because there was no a priori evidence that the concepts we had selected, based on models and questionnaires mostly developed and validated in Western populations, would apply to other cultures, languages, and health systems [14, 15]. The simultaneous development of French and US English versions helped maximize the cultural and conceptual equivalence of the tool and allowed possible future issues during translation to other languages from the same linguistic group to be anticipated and resolved. The supportive results of qualitative testing in Mandarin provided a confirmation of the universality of the SPUR framework and of the wording of the SPUR tool. Our findings showed that the Chinese and the UK versions of the tool were successfully translated from the US version. The US version will therefore be used to support communication and as a master version for other future translations. The availability of these different versions will facilitate its use in European, American, and Chinese studies. However, additional cross-cultural research is needed in order to confirm the validity of the measurement framework in terms of concept, wording, and response, before conducting psychometric equivalence tests [16].

Beyond the specific aim of providing a tool for clinicians to identify patient-level drivers of medication adherence, the patient responses given during the cognitive interviews also provided an opportunity to evaluate the wider benefits of the assessment. While responding to the assessment items, patients described their daily medication-taking and self-management habits within their social environment, their beliefs regarding their interactions with the medical team, and the way they believed their action would have an impact on their own health. These discussions resulted in patients from all countries spontaneously commenting that the SPUR tool helped them to take a step back regarding their understanding of their condition, and helped them realize that other people were sharing the same events in their daily lives. Some patients also asked the interviewers for feedback regarding their own situation as compared to others.

How is SPUR different compared to other tools? First, none of the previously developed tools offer a comprehensive coverage of all SPUR factors. Second, SPUR is applicable to patients taking any medication type for a variety of chronic conditions; in contrast many other adherence measures are disease or treatment specific. Third, SPUR focuses on determinants of adherence, contrary to instruments such as the Morisky Medication Adherence Scale (MMAS) which measure the actual level of adherence [17]. Other generic tools aim at capturing the determinants of adherence to treatment in chronic conditions, such as Accept [18]. However, while Accept captures the treatment characteristics, SPUR considers factors measured at the level of the patient. Last but not least, while most of the existing instruments aim at being used for research or evaluation, SPUR aims at providing individualized profiles to help decisions about the need for a behavioral intervention, and to help guide such an intervention if needed, and tailor it to the patient’s needs.

A potential limitation of the development process of the SPUR tool was that patients were not involved in the initial development phase. However, the items included in the tool were generated after evaluation of existing measures and patient feedback was then used to effectively optimize the SPUR tool. More generally, one of the main limitations of adherence tools is that they are particularly exposed to social desirability bias [19, 20]. However, in the case of the SPUR tool, we aimed to limit this risk of bias by deliberately avoiding including items related to treatment compliance (“how many times did you take your medication”, etc.). We also payed specific attention to how items were worded, ensuring questions were posed in a non-judgmental way. Results from the cognitive interviews conducted did not reveal concerns related to social desirability bias. Although this initial phase of development of the SPUR tool provided encouraging results concerning its clinical relevance across cultures and in a wide range of patients with different chronic diseases, the tool may still have some limitations. Health care systems in some countries vary considerably between regions and although our study indicated that the SPUR tool could be adapted for use in different languages and countries, the data collected were insufficient to assess whether regional variations in health care had any impact on the acceptability of the tool. In addition, the issues surrounding adherence are often specific to each medication prescribed, the disease being treated and the patient’s history [21]. It is therefore possible that some aspects of issues surrounding particular types of medication for some diseases or comorbidities are not covered by the SPUR tool. In particular, further exploration of the responses of patients with multiple conditions needs be considered, in order to ensure that patients with multiple morbidities are able to respond in a valid and reliable way.

In addition to these further studies, the next steps will be the determination of the tool’s scoring, psychometric properties and its validation via examining correlations with other self-reported adherence measures and objective measures of medication use (such as blood levels), and longitudinal changes in health status. These same studies will investigate the possibility of using Computerized Adaptive Testing administration in a digital environment in order to tailor the questionnaire to individual patients.

## Conclusions

The SPUR tool is an innovative self-reported measure of risks of nonadherence for patients taking long-term treatments. Its development methodology based on qualitative methods provides promising evidence of its validity across cultural settings and conditions. The next steps will be designed to confirm this hypothesis in larger quantitative studies.

## Supplementary Information

**Additional file 1: **Interview Guide.

**Additional file 2: **Instrument Review.

**Additional file 3: **Detailed results of the two rounds of cognitive interviews of the US English version.

**Additional file 4: **SPUR Tool Pilot Version.

## Data Availability

The datasets used and/or analyzed during the current study are available from the corresponding author on reasonable request.

## Abbreviations

COPD: Chronic obstructive pulmonary disease
HBM: Health Belief Model
IAB: International Advisory Board
NRS: Numerical rating scale
SPUR: Social, Psychological, Usage, Rationale
TPB: Theory of Planned Behavior
