# Supplementary material for: Development of the SPUR tool: a profiling instrument for patient treatment behavior
Source: J Patient Rep Outcomes. 2022 Jun 6;6:61. doi: 10.1186/s41687-022-00470-x (PMC9170867; doi:10.1186/s41687-022-00470-x)
Supplement: Supplementary file 1 — Additional file 1: Interview Guide. [file 41687_2022_470_MOESM1_ESM.docx]

# **Introduction to the interview**

1. **Introduction & objective(s)**

- Check that the participant has been duly informed
- Thank the participant for agreeing to participate in the interview.
- Introduce [Company name]: a company that develops health questionnaires for patients
- Introduce the questionnaire: questionnaire designed to understand patients’ treatment-related beliefs and habits
- Explain the objective(s) of the interview: to check that the questionnaire is easily understood by people on long-term treatment

1. **Confidentiality**

- Remind the participant that:
- The interview will be analyzed in a way that protects participant’s privacy by avoiding any mention of their name or any other identifying information (e.g.: name, place…);
- What the participant says will be analyzed along with information from others participating in the study.

1. **Structure of the interview and recording**

- Remind the participant about:
- Duration of the interview: one hour;
- Recording of the interview to facilitate the discussion;
- Confidentiality of the recording: being accessible only to the people in charge of analysis;
- Destruction of the recording at the end of the study;
- Their rights to interrupt the interview at any time and/or to not answer a question;
- Freedom in their answers: being opened in their responses and not being afraid to share what they think.
- Inform the participant that some questions might be repetitive but are necessary to well capture their opinion about the questionnaire.

1. **Questions**

- Check the participant accepts the interview
- Check the participant has a copy of the questionnaire nearby (print or pdf on a computer, smartphone, tablet, etc.)
- Give the participant the opportunity to ask any questions before starting the interview.

1. **Start recording**

- State:
- Date
- Participant’s identification number.
- Obtain and record participant consent for recording the interview.

# **Reading of the questionnaire**

- Ask the participant whether they have read the questionnaire before the interview:
- If yes, give them the opportunity to read it again
- If no, ask them to take the time to read it now
- If possible, record the time it takes the participant to read the questionnaire.

# **Cognitive interview**

Note to the interviewer:

- Below are listed themes/concepts (preceded by 🗣/o) to be explored during interviews. These themes/concepts should be introduced by the interviewer to the interviewee with language and wording appropriate to the level of understanding of the interviewee.
- Probes are written in *italics* and are to be used only when necessary (i.e., if a participant experiences difficulties in spontaneously answering a question or to collect additional information to further explore a concept of interest).
- Open-ended questions should be used as much as possible with close-ended questions used to clarify or elicit precise information.
- Encourage the participant to give examples to better illustrate the situation or description.
- Leave enough time for the participant to full report their thoughts.

## General impressions

- Overall opinion of the *questionnaire*
  - *Difficult level:*
  - *Readability*
  - *Comprehension*
  - *Suggestions on how to make it easier*
- Overall opinion of the general appearance (format / layout) of the questionnaire
- Opinion about the length of the *questionnaire*
- Question order
- Add questions (if yes, ask which ones)
- Delete questions (if yes, ask which ones)

## Title and instructions

*The Observia Treatment Behavior Questionnaire:*

- Title meaning
- Title make sense
- Suggestions on changes to the title

*Instructions:*

- Instructions: ask if they read, skimmed, or skipped instructions
- Instruction meaning
- Add anything to the instructions
- Suggestions on changes to the instructions

## Questions and responses choices

Note to the interviewer:

- For multiple items*,* **clearly state the item number each time.**
- For each item*,* ask the same set of questions (i.e. ‘Question to be tested for all items’ – see below) plus item-specific questions, including possible of alternative wording (see table below).
- For items with the consistency comment, please ensure that participant’s answers on the scale are matching their opinion about the statement of the item
- Ex: item #36 “I don't like taking medications”: if the participant explains that he/she ‘likes’ (or doesn’t mind) taking medications, he/should have checked one of the boxes on the left side (near “I strongly disagree”) of the response scale; if they check the boxes closer to the right side, there is an inconsistency that should be probed.
- Encourage the participant to give examples, to illustrate their answers.
- When proposing an alternative wording of the items, please ask the participant which one they prefer and why.
- [Health problem] is adapted to each participant’s condition, i.e. diabetes, multiple sclerosis or breast cancer.

**Question to be tested for all items:** Items 1-49

- Meaning/understanding
- Relevance according to experience
- Suggestion to reword
- Add or remove questions (if yes, ask why?)

**Answer choices to be tested:** 10-point Likert scale

- Define scale in your own words (ask once with question #1)
- Response choices/scale suitable for the question
- Suggestions on changes to the response choices/scale

| **Item #** | **Item wording - Test version 3.0 01-JUN-2018** | **Item-specific question** |
| --- | --- | --- |
| 1 | My [health problem] affects my relationships with those I care about. | - Meaning/example(s) of ‘relationships’ |
| 2 | My [health problem] affects my social life. | - Meaning/example(s) of ‘social life’ |
| 3 | The fact that others with [my health problem] follow their treatment plan helps me follow mine. | - Meaning/example(s) of ‘treatment plan’ vs. ‘treatment’ |
| 4 | Most people with [my health problem] would follow their treatment plan precisely. | - Meaning/examples(s) of ‘precisely’ |
| 5 | My loved ones help me manage my [health problem]. |  |
| 6 | Other people in my life play a big part in whether I feel healthy or my [health problem] gets worse. | - Meaning/example(s) of ‘healthy’ - Alternative wording: Other people play a big role in whether my [health problem] improves, stays the same, or gets worse |
| 7 | Staying healthy is very important to me. |  |
| 8 | Precisely following health care providers’/doctors’ recommendations is the best way for me to stay healthy. | - Meaning/example(s) of ‘health care providers’ - Alternative wording: - Health care providers/doctors help keep me healthy - I trust my healthcare provider's recommendations |
| 9 | Following my treatment plan is not up for discussion, I have to do it. | - Meaning/example(s) of ‘up for discussion’ - Check consistency between item comprehension and response choice - Alternative wording: It is essential that I follow my treatment plan |
| 10 | Sometimes it seems to me that my [health problem] just isn't real. |  |
| 11 | I'm the kind of person who will follow their treatment plan precisely. |  |
| 12 | I feel that my [health problem] is something that I won’t have to live with for the rest of my life. | - Alternative wording: I feel that my [health problem] is something that I will have to live with for the rest of my life |
| 13 | I focus more on the present than on the future. | - Meaning/example(s) of ‘future’ - Alternative wording: I tend to focus on the present and do not worry about the future |
| 14 | If my doctor tells me to do something, I do it. |  |
| 15 | Sometimes doctors prescribe treatment you don't really need. |  |
| 16 | Sometimes I don't follow my treatment plan precisely. | - Check consistency between item comprehension and response choice |
| 17 | I find it easy to get my treatment for my [health problem]. | - Alternative wording: I find it easy to get my prescriptions/medications for my [health problem] - Difference between ‘medication’ and ‘treatment’ |
| 18 | I don’t have any trouble paying for my treatment. | - Meaning/example(s) of ‘trouble paying’ - Check consistency between item comprehension and response choice |
| 19 | I am able to follow my treatment plan without much difficulty. |  |
| 20 | The doctors and other health professionals sometimes ignore what I tell them. | - Alternative wording: Doctors and other health professionals don't listen to what I have to say |
| 21 | My [health problem] has led to financial problems. |  |
| 22 | I find it easy to organize my treatment plan. | - Alternative wording: I find it easy to organize the treatment for my [health problem] |
| 23 | I find it easy to follow my treatment plan when I am not at home. | - Meaning/example(s) of ‘being not at home’ |
| 24 | I am satisfied with the level of information I have about my treatment. | - Meaning/example(s) of ‘level of information |
| 25 | I find it easy to manage the different medications I take. |  |
| 26 | I find it easy to take my medication for my [health problem]. |  |
| 27 | I am worried about the side effects of some treatments. | - Meaning/example(s) of ‘side effects’ |
| 28 | Medications should only be taken when needed. |  |
| 29 | I have no control over my [health problem]. | - Meaning/example(s) of ‘control’ - Check consistency between item comprehension and response choice - Alternative wording: No matter what I do, my [health problem] will get worse |
| 30 | I am concerned about becoming too dependent on my treatment. | - Meaning/example(s) of ‘becoming too dependent’ |
| 31 | I am worried about taking medications. |  |
| 32 | My [health problem] is very severe. | - Meaning/example(s) of ‘very severe’ |
| 33 | My [health problem] does not affect my ability to exercise. | - Meaning/example(s) of ‘exercise’ - Check consistency between item comprehension and response choice - Alternative wording: I am able to get physical activity in spite of my [health problem] |
| 34 | My treatment affects my sex life. |  |
| 35 | I am satisfied with the level of information I have about my [health problem]. | - Alternative wording: I completely understand my [health problem] |
| 36 | I don't like taking medications. | - Check consistency between item comprehension and response choice |
| 37 | Medications are not good for me. | - Check consistency between item comprehension and response choice - Alternative wording: Medications don't do anything for me |
| 38 | My treatment helps my [health problem] to improve. |  |
| 39 | There is no point in taking medications for my [health problem]. | - Meaning/example(s) of ‘no point’ - Check consistency between item comprehension and response choice |
| 40 | My [health problem] treatment will keep me healthy. | - Alternative wording: My [health problem] medication will keep me healthy |
| 41 | My actions directly affect my [health problem]. | - Meaning/example(s) of ‘actions’ - Alternative wording: I am in control of my health |
| 42 | My [health problem] is unlikely to get worse whether I follow my treatment plan or not. | - Meaning/example(s) of ‘get worse’ |
| 43 | I feel worse if I don't follow my treatment plan. | - Alternative wording: I feel worse if I don't take my medication |
| 44 | Medications are more expensive than they should be. |  |
| 45 | My [health problem] keeps me from doing things I want to do. | - Meaning/example(s) of ‘things’ |
| 46 | Following my [health problem] treatment plan lets me do the things I want to do. |  |
| 47 | I focus better when I am on my treatment plan. | - Meaning/example(s) of ‘focus’ - Alternative wording: I find it easy to concentrate when I follow my treatment plan |
| 48 | I believe non-traditional treatments can replace some of my medications. | - Meaning/example(s) of ‘non-traditional treatments’ |
| 49 | I have found ways to deal with my [health problem]. |  |

# **End of the interview**

## Collection of socio-demographic information

- Please ask the following socio-demographic information to the participant.
- Age
- Gender:
- Male
- Female
- Highest level of education (please check one):
- Some high school
- High school
- Bachelor’s degree
- Post-graduate degree
- Other, please specify: _____________________
- Current work status (please check one):
- Full-time
- Part-time
- Student
- Retired
- Unemployed

## Conclusion

- Ask the participant if they would like to add anything.
- Thank the participant for participating in the interview.
- Stop the recording equipment and check the recording. In the event that the interview was not recorded, please write extensive notes based on your recent memory. Please also note the duration of the interview.
