# Supplementary material for: Development of the SPUR tool: a profiling instrument for patient treatment behavior
Source: J Patient Rep Outcomes. 2022 Jun 6;6:61. doi: 10.1186/s41687-022-00470-x (PMC9170867; doi:10.1186/s41687-022-00470-x)
Supplement: Supplementary file 2 — Additional file 2: Instrument Review. [file 41687_2022_470_MOESM2_ESM.docx]

# Instrument review

| Acronym | Instrument full name | Objective | # items | Domain(s) |
| --- | --- | --- | --- | --- |
| AACTG ([Chesney et al. 2000](#_ENREF_4)) | Adult AIDS Clinical Trials Group adherence questionnaire | To measure recent adherence by minimizing bias and maximizing recall | 6 | Adherence ratios How closely schedule followed? How closely instructions followed? When any medication last skipped? Whether any medication missed last weekend? Reasons for missed medication |
| ADQ ([DiMatteo et al. 1993](#_ENREF_7)) | Adherence Determinants Questionnaire | To assess elements of patients' adherence to medical treatment and prevention | 38 | Interpersonal aspects of care Perceived utility  Perceived severity  Perceived susceptibility  Subjective norms  Intentions  Supports/barriers |
| AHBI ([Park et al. 2015](#_ENREF_19)) | Health Belief Model-the arthritis-related health belief instrument | To assess individual perceptions of nonpharmacological pain therapy among and measure likelyhood of nonpharmacological pain therapy in older adults. | 16 | Severity  Benefits  Barriers  Susceptibility |
| BAASIS ([Dobbels et al. 2010](#_ENREF_8)) | Basel Assessment of Adherence to Immunosuppressive Medications Scale | To assess adherence to immunosuppressive medication in adults transplant patients | 4 | Dose taking Drug holidays Timing deviation >2 h from prescribed time Dose reduction |
| B-IPQ ([Broadbent et al. 2006](#_ENREF_2)) | Brief Illness Perception Questionnaire | To rapidly assess the cognitive and emotional representations of illness | 9 | Consequences Timeline Personal control Treatment control Identity Concern Understanding Emotional responses Causal representation |
| BMQ ([Horne et al. 1999](#_ENREF_15)) | Beliefs about Medicines | To assess cognitive representations of medication. The BMQ-Specific assesses representations of medication prescribed for personal use and the BMQ-General assesses beliefs about medicines in general | 18 | BMQ-Specific  -Specific-Necessity: beliefs about the necessity of prescribed medication -Specific-Concerns: concerns about prescribed medication based on beliefs about the danger of dependence and long-term toxicity and the disruptive effects of medication BMQ-General -General-Harm: beliefs that medicines are harmful, addictive, poisons which should not be taken continuously -General-Overuse: beliefs that medicines are overused by doctors |
| BMQ1 ([Svarstad et al. 1999](#_ENREF_22)) | Brief Medication Questionnaire | To screen for adherence and barriers to adherence | 9 | Regimen Screen: asks patients how they took each medication in the past week  Belief Screen: asks about drug effects and bothersome features  Recall Screen: about potential difficulties remembering |
| BP-Self-Care ([Peters and Templin 2008](#_ENREF_20)) | Blood Pressure Self-Care Scale | To measure self-care practices regarding behaviors needed for blood pressure control | 10 | No named domains |
| CHBMS ([Champion 1984](#_ENREF_3)) | Champions health belief model scale | To assess health beliefs and perceptions about screening | 36 | Perceived susceptibility  Perceived seriousness  Perceived benefits  Perceived barriers  Health motivation  Confidence |
| CQR ([de Klerk et al. 2003](#_ENREF_6)) | Compliance Questionnaire for Rheumatology | To assess patient compliance rheumatoid arthritis, polymyalgia rheumatica, and gout. | 19 | No named domains |
| DAI ([Hogan et al. 1983](#_ENREF_12)) | Drug Attitude Inventory | To measure subjective responses and attitudes towards maintenance of antipsychotic drug therapy | 10- & 30-item versions | Subjective positive  Subjective negative  Health and illness  Physicians  Control issues  Prevention  Harm |
| GKQ ([Spaetgens et al. 2016](#_ENREF_21)) | Gout Knowledge Questionnaire | To assess gout patients regarding their level of disease-related knowledge in order to identify potential targets for patient education. | 10 | Disease knowledge |
| GTCAT ([Barker et al. 2015](#_ENREF_1)) | Glaucoma Treatment Compliance Assessment Tool | To evaluate health behavior and glaucoma adherence | 47 | Barriers  Benefits  Cues-to-action  Knowledge  Patient-physician relationship  Self-efficacy  Self-report adherence  Severity  Susceptibility |
| IPQ ([Weinman et al. 1996](#_ENREF_26)) | Illness Perceptions Questionnaire | To assess cognitive representations of illness | 38 | Identity scale Timeline  Consequences  Cure control  Cause |
| IPQ-R ([Moss-Morris et al. 2002](#_ENREF_18)) | Revised Illness Perception Questionnaire | To assess cognitive representations of illness | 70 | Identity  Timeline (acute/chronic)  Timeline (cyclical)  Consequences  Personal control  Treatment control  Emotional representations  Illness coherence  Psychological attributions  Risk factor attributions  Immune attributions  Chance attributions |
| LMQ ([Krska et al. 2014](#_ENREF_16)) | Living with Medicines Questionnaire | To measure overall medicines burden | 42 | Patient–doctor relationships and communication about medicines  Interferences with daily life  Practicalities  Effectiveness  Patient–pharmacist communication about medicines  Acceptance of medicine use  Autonomy/control over medicine use  Concerns about potential harm |
| MARS ([Thompson et al. 2000](#_ENREF_23)) | Medication Adherence Rating Scale | To measure medication compliance with psychiatric patients | 10 | Medication adherence behaviour  Subjects’ attitude to taking medication  Negative side-effects and attitudes to psychotropic medication |
| MARS-A ([Cohen et al. 2009](#_ENREF_5)) | Medication Adherence Report Scale for Asthma | To measure adherence with inhaled corticosteroids among patients with persistent asthma | 10 | No named domains |
| MDKT ([Fitzgerald et al. 1998](#_ENREF_9)) | Michigan diabetes knowledge test | To assess diabetes knowledge of patients | 23 | General disease knowledge Insulin knowledge |
| MHLC ([Wallston et al. 1978](#_ENREF_24)) | Multidimensional Health Locus of Control Scales | To assess individuals’ feelings of control over their illness or disease | 18 | Internality Chance externality Powerful others externality |
| MMAS ([Morisky et al. 2008](#_ENREF_17)) | Morisky Medication Adherence Scale | To assess patient medication-taking behavior | 4- & 8-item versions | No named domains |
| MRCI ([George et al. 2004](#_ENREF_11)) | Medication Regimen Complexity Index | To quantify medication regimen complexity at the patient level | 65 | Dosage form Dosing frequency Additional administration instructions |
| PD-Rx ([Fleisher et al. 2016](#_ENREF_10)) | Parkinson's Disease Medication Beliefs Scale | To elicit medication beliefs of persons with Parkinson's disease | 11 | Motor improvement/self-efficacy Current adverse effects Future concerns |
| PSM ([Horne et al. 2013](#_ENREF_13)) | Perceived Sensitivity to Medicines Scale | To assess perceptions of perceived sensitivity to potential adverse effects of medicines | 5 | Perceived sensitivity |
| RAM ([Horne et al. 1999](#_ENREF_15)) | Reported Adherence Scale | To assess non-adherence as the tendency to forget to take medication and to deliberately adjust or alter the dose from that recommended by the physician | 4 | No named domains |
| SIMS ([Horne et al. 2001](#_ENREF_14)) | Satisfaction with Information about Medicines Scale | To assess the extent to which patients feel they have received enough information about prescribed medicines | 17 | Action and usage Potential problems of medication |
| TES ([Webb et al. 2001](#_ENREF_25)) | Treatment Empowerment Scale | To assess patients’ perceptions of empowerment in the context of drug therapy | 10 | Communication Treatment choice Decision-making  Satisfaction |

References:

Barker, G. T., Cook, P. F., Schmiege, S. J., Kahook, M. Y., Kammer, J. A., & Mansberger, S. L. (2015). Psychometric properties of the Glaucoma Treatment Compliance Assessment Tool in a multicenter trial. *Am J Ophthalmol, 159*(6), 1092-1099 e1092, doi:10.1016/j.ajo.2015.03.006.

Broadbent, E., Petrie, K. J., Main, J., & Weinman, J. (2006). The brief illness perception questionnaire. *J Psychosom Res, 60*(6), 631-637, doi:10.1016/j.jpsychores.2005.10.020.

Champion, V. L. (1984). Instrument development for health belief model constructs. *ANS Adv Nurs Sci, 6*(3), 73-85.

Chesney, M. A., Ickovics, J. R., Chambers, D. B., Gifford, A. L., Neidig, J., Zwickl, B., et al. (2000). Self-reported adherence to antiretroviral medications among participants in HIV clinical trials: the AACTG adherence instruments. Patient Care Committee & Adherence Working Group of the Outcomes Committee of the Adult AIDS Clinical Trials Group (AACTG). *AIDS Care, 12*(3), 255-266, doi:10.1080/09540120050042891.

Cohen, J. L., Mann, D. M., Wisnivesky, J. P., Home, R., Leventhal, H., Musumeci-Szabo, T. J., et al. (2009). Assessing the validity of self-reported medication adherence among inner-city asthmatic adults: the Medication Adherence Report Scale for Asthma. *Ann Allergy Asthma Immunol, 103*(4), 325-331.

de Klerk, E., van der Heijde, D., Landewe, R., van der Tempel, H., & van der Linden, S. (2003). The compliance-questionnaire-rheumatology compared with electronic medication event monitoring: a validation study. *J Rheumatol, 30*(11), 2469-2475.

DiMatteo, M. R., Hays, R. D., Gritz, E. R., Bastani, R., Crane, L., Elashoff, R., et al. (1993). Patient adherence to cancer control regimens: Scale development and initial validation. *Psychological Assessment, 5*(1), 102-112, doi:10.1037/1040-3590.5.1.102.

Dobbels, F., Berben, L., De Geest, S., Drent, G., Lennerling, A., Whittaker, C., et al. (2010). The psychometric properties and practicability of self-report instruments to identify medication nonadherence in adult transplant patients: a systematic review. *Transplantation, 90*(2), 205-219, doi:10.1097/TP.0b013e3181e346cd.

Fitzgerald, J. T., Funnell, M. M., Hess, G. E., Barr, P. A., Anderson, R. M., Hiss, R. G., et al. (1998). The reliability and validity of a brief diabetes knowledge test. *Diabetes Care, 21*(5), 706-710, doi:10.2337/diacare.21.5.706.

Fleisher, J. E., Dahodwala, N. A., Xie, S. X., Mayo, M., Weintraub, D., Chodosh, J., et al. (2016). Development and Validation of the Parkinson's Disease Medication Beliefs Scale (PD-Rx). *J Parkinsons Dis, 6*(2), 383-392, doi:10.3233/jpd-150765.

George, J., Phun, Y. T., Bailey, M. J., Kong, D. C., & Stewart, K. (2004). Development and validation of the medication regimen complexity index. *Ann Pharmacother, 38*(9), 1369-1376, doi:10.1345/aph.1D479.

Hogan, T. P., Awad, A. G., & Eastwood, R. (1983). A self-report scale predictive of drug compliance in schizophrenics: reliability and discriminative validity. *Psychol Med, 13*(1), 177-183.

Horne, R., Faasse, K., Cooper, V., Diefenbach, M. A., Leventhal, H., Leventhal, E., et al. (2013). The perceived sensitivity to medicines (PSM) scale: an evaluation of validity and reliability. *Br J Health Psychol, 18*(1), 18-30, doi:10.1111/j.2044-8287.2012.02071.x.

Horne, R., Hankins, M., & Jenkins, R. (2001). The Satisfaction with Information about Medicines Scale (SIMS): a new measurement tool for audit and research. *Qual Health Care, 10*(3), 135-140, doi:10.1136/qhc.0100135..

Horne, R., Weinman, J., & Hankins, M. (1999). The beliefs about medicines questionnaire: The development and evaluation of a new method for assessing the cognitive representation of medication. *Psychol Health, 14*(1), 1-24, doi:10.1080/08870449908407311.

Krska, J., Morecroft, C. W., Rowe, P. H., & Poole, H. (2014). Measuring the impact of long-term medicines use from the patient perspective. *Int J Clin Pharm, 36*(4), 675-678, doi:10.1007/s11096-014-9970-5.

Morisky, D. E., Ang, A., Krousel-Wood, M., & Ward, H. J. (2008). Predictive validity of a medication adherence measure in an outpatient setting. *J Clin Hypertens (Greenwich), 10*(5), 348-354.

Moss-Morris, R., Weinman, J., Petrie, K., Horne, R., Cameron, L., & Buick, D. (2002). The Revised Illness Perception Questionnaire (IPQ-R). *Psychol Health, 17*(1), 1-16, doi:10.1080/08870440290001494.

Park, J., Clement, R., Hooyman, N., Cavalie, K., & Ouslander, J. (2015). Factor structure of the Arthritis-Related Health Belief instrument in ethnically diverse community-dwelling older adults with chronic pain. *J Community Health, 40*(1), 73-81, doi:10.1007/s10900-014-9898-7.

Peters, R. M., & Templin, T. N. (2008). Measuring blood pressure knowledge and self-care behaviors of African Americans. *Res Nurs Health, 31*(6), 543-552, doi:10.1002/nur.20287.

Spaetgens, B., Pustjens, T., Scheepers, L., Janssens, H., van der Linden, S., & Boonen, A. (2016). Knowledge, illness perceptions and stated clinical practice behaviour in management of gout: a mixed methods study in general practice. *Clin Rheumatol, 35*(8), 2053-2061, doi:10.1007/s10067-016-3212-2.

Svarstad, B. L., Chewning, B. A., Sleath, B. L., & Claesson, C. (1999). The Brief Medication Questionnaire: a tool for screening patient adherence and barriers to adherence. *Patient Educ Couns, 37*(2), 113-124.

Thompson, K., Kulkarni, J., & Sergejew, A. A. (2000). Reliability and validity of a new Medication Adherence Rating Scale (MARS) for the psychoses. *Schizophr Res, 42*(3), 241-247.

Wallston, K. A., Wallston, B. S., & DeVellis, R. (1978). Development of the Multidimensional Health Locus of Control (MHLC) Scales. *Health Educ Monogr, 6*(2), 160-170.

Webb, D. G., Horne, R., & Pinching, A. J. (2001). Treatment-related empowerment: preliminary evaluation of a new measure in patients with advanced HIV disease. *Int J STD AIDS, 12*(2), 103-107, doi:10.1258/0956462011916875.

Weinman, J., Petrie, K. J., Moss-morris, R., & Horne, R. (1996). The illness perception questionnaire: A new method for assessing the cognitive representation of illness. *Psychol Health, 11*(3), 431-445, doi:10.1080/08870449608400270.
