# Supplementary material for: Development of the SPUR tool: a profiling instrument for patient treatment behavior
Source: J Patient Rep Outcomes. 2022 Jun 6;6:61. doi: 10.1186/s41687-022-00470-x (PMC9170867; doi:10.1186/s41687-022-00470-x)
Supplement: Supplementary file 3 — Additional file 3: Detailed results of the two rounds of cognitive interviews of the US English version. [file 41687_2022_470_MOESM3_ESM.docx]

# Detailed results of the two rounds of cognitive interviews of the US English version of the whole questionnaire

| **SPUR-Test-1 (round 1)** | | | | | **SPUR-Test-2 (round 2)** | | | | | **SPUR-Pilot** | | |
| --- | --- | --- | --- | --- | --- | --- | --- | --- | --- | --- | --- | --- |
| **Items** | | | Results | Decision | **Items** | | | Results | Decision | **Items** | | |
| **S** | **1** | **My [health problem] affects my relationships with those I care about.** | Item understood and found relevant. | No change | **S** | **1** | **My [health problem] affects my relationships with those I care about.** | Item understood, found relevant and “*easy to answer*”. | No change | **S** | **1** | **My [health problem] affects my relationships with those I care about.** |
| **S** | **2** | **My [health problem] affects my social life.** | Item understood and found relevant | No change | **S** | **2** | **My [health problem] affects my social life.** | Item understood, found relevant and “*easy to answer*”. | No change | **S** | **2** | **My [health problem] affects my social life.** |
| **S** | **3** | **The fact that others with [my health problem] follow their treatment plan helps me follow mine.** | Item understood but 3 patients did not find it relevant because they didn't care what anyone else did. | Reworded | **S** | **3** | **If others with [health problem] follow their treatment plan, I don't care.** | Item understood but confusing. In general, patients don't care what others do. | Reworded | **S** | **3** | **I would be interested in knowing if others with [health problem] follow their treatment plan.** |
| **S** | **4** | **Most people with [my health problem] would follow their treatment plan precisely.** | Item understood.  4/7 patients did not like the word "*precisely*", "*because it was putting a burden on them*" according to one of them.  1 patient suggested to change to "exactly". | Reworded | **S** | **4** | **Most people with [health problem] would follow their doctors' prescription exactly.** | Like item #3, relevance varied from patient to patient. Moreover, "most people" was confusing for some of them. | Reworded | **S** | **4** | **I think that people with [health problem] generally follow their doctors' prescription exactly.** |
| **S** | **5** | **My loved ones help me manage my [health problem].** | Item understood and found relevant, but 1 patient thought this question wouldn't apply to everyone as some people don't have any "loved ones". | Merged | **S** | **5** | **Other people in my life help me manage my [health problem].** | Item understood and found relevant. However, "other people" was qualified as "*vague*", "*ambiguous*" or "*confusing*". | Reworded | **S** | **5** | **The people in my life help me manage my [health problem].** |
| **S** | **6** | **Other people in my life play a big part in whether I feel healthy or my [health problem] gets worse.** | Item understood; most patients found it relevant, but 4 patients suggested rewording the item, including 1 patient who did not like the negative connotation. |  |  |  |  |  |  |  |  |  |
| **P** | **7** | **Staying healthy is very important to me.** | Item understood and found relevant: "*relevant and straightforward*", "*fine*", "*excellent question*".  A French patient answered: “*It’s important but we don’t choose*”. | Reworded | **P** | **6** | **Fighting for my health is my highest priority.** | Item understood and found relevant. | No change | **P** | **6** | **Fighting for my health is my highest priority.** |
| **P** | **8** | **Precisely following health care providers’/doctors’ recommendations is the best way for me to stay healthy.** | Item understood. 1 patient asked to remove "health care providers". | Reworded | **P** | **7** | **Precisely following doctors’ recommendations is the best way for me to stay healthy.** | Item understood and found relevant, but 4 patients preferred the alternative "*I trust doctors' recommendations*"; 1 patient liked both propositions and found them different. | No change | **P** | **7** | **Precisely following doctors’ recommendations is the best way for me to stay healthy.** |
|  |  |  |  |  |  |  |  |  | Alternative added | **P** | **8** | **I trust doctors' recommendations.** |
| **P** | **9** | **Following my treatment plan is not up for discussion, I have to do it.** | Item understood and found relevant. 2 patients preferred the alternative wording "*It is essential that I follow my treatment plan*" | Reworded | **P** | **8** | **It is essential that I follow my treatment plan.** | Item understood and found relevant "*for a questionnaire like this*", "*straight to the point*". | No change | **P** | **9** | **It is essential that I follow my treatment plan.** |
| **P** | **10** | **Sometimes it seems to me that my [health problem] just isn't real.** | Item understood and found relevant. | No change | **P** | **9** | **Sometimes it seems to me that my [health problem] just isn't real.** | Rather relevant but there were different interpretations. | Reworded | **P** | **10** | **Sometimes my [health problem] seems unreal to me.** |
| **P** | **11** | **I'm the kind of person who will follow their treatment plan precisely.** | Item understood and mostly found relevant. 1 patient found it repetitive, and 2 patients suggested to replace "precisely" by "exactly". | Reworded | **P** | **10** | **I'm the kind of person who will follow their treatment plan exactly.** | Item understood and found relevant. | No change | **P** | **11** | **I'm the kind of person who will follow their treatment plan exactly.** |
| **P** | **12** | **I feel that my [health problem] is something that I won’t have to live with for the rest of my life.** | Item understood and mostly found relevant. 4/7 patients preferred the positive connotation of the alternative "*I feel that my [health problem] is something that I will have to live with for the rest of my life*" and 1 patient suggested to remove "*I feel that*" and start with "*My [health problem]*". | Split  1^st^ item: treatment duration  2^nd^ item: illness duration | **P** | **11** | **I would have to take a treatment for my [health problem] for the rest of my life.** | Item understood and found relevant but conditional tense was confusing: 3 patients suggested to replace "would" by "will". | Reworded | **P** | **12** | **I will have to take a treatment for my [health problem] for the rest of my life.** |
|  |  |  |  |  | **P** | **12** | **My [health problem] can be cured.** | Item understood but viewed differently among patients: either "*depressing*" or "*hopeful*", "*not too relevant*", "*no cure for these diseases*". | Deleted |  |  |  |
| **P** | **13** | **I focus more on the present than on the future.** | Item understood by 6/7 patients and found relevant by 4/7 patients. However, French patients underlined there were 2 parts in the question: 1) Focus on present, 2) Not worry about the future. | Reworded | **P** | **13** | **I live for the present.** | Item mostly understood (5/8 patients) but also perceived as "*useless*" and "*irrelevant*".  1 patient thought it needed to be more specific. | Reworded | **P** | **13** | **I live in the moment.** |
| **P** | **14** | **If my doctor tells me to do something, I do it.** | Item understood and found relevant.  2 patients thought it was repetitive. | Reworded | **P** | **14** | **If my doctor recommends me to do something, I do it.** | Item understood and found relevant. | Reworded (grammar corrected) | **P** | **14** | **If my doctor recommends that I do something, I do it.** |
| **P** | **15** | **Sometimes doctors prescribe treatment you don't really need.** | Item understood; 3/7 patients found it relevant and 1 patient thought it was “*assumptuous*”. | No change | **P** | **15** | **Sometimes doctors prescribe treatment you don't really need.** | Item understood. Relevance depended on the disease: patients with breast cancer found this item "irrelevant to cancer patients", "inappropriate for breast cancer". | No change | **P** | **15** | **Sometimes doctors prescribe treatment you don't really need.** |
| **P** | **16** | **Sometimes I don't follow my treatment plan precisely.** | Item understood. However, 2 patients were struggled with the use of the word "precisely." and 1 patient suggested using the word "exactly". 2 patients thought it was repetitive, but another patient thought it was "*good to double check patients’ true opinion by asking the same question in different ways*". | Reworded | **P** | **16** | **Sometimes I don't follow my treatment plan exactly.** | Item understood and mostly found relevant, "*appropriate*". | No change | **P** | **16** | **Sometimes I don't follow my treatment plan exactly.** |
| **U** | **17** | **I find it easy to get my treatment for my [health problem].** | Item understood. Patients were able to differentiate between treatment and medication, saying one was a broader term. | No change | **U** | **17** | **I find it easy to get my treatment for my [health problem].** | Item understood and found relevant. | No change | **U** | **17** | **I find it easy to get my treatment for my [health problem].** |
| **U** | **18** | **I don’t have any trouble paying for my treatment.** | Item understood and relevant: "*It’s a good question if they wanna see who can and cannot pay for their treatment*". 1 patient said to underline the "don't". | Reworded  to align with French wording and avoid the negative form | **U** | **18** | **It is easy for me to pay for my treatment.** | Item understood and found relevant.  5/8 patients preferred the alternative "*I can easily pay for my treatment*". | Reworded | **U** | **18** | **I can easily pay for my treatment.** |
| **U** | **19** | **I am able to follow my treatment plan without much difficulty.** | Item understood and found relevant. 1 patient thought "much difficulty" was a "*quantitative phrase*" and said "*you either have a little difficulty or a lot of difficulty*". | Reworded | **U** | **19** | **I am able to follow my treatment plan.** | Item understood and found relevant. | No change | **U** | **19** | **I am able to follow my treatment plan.** |
| **U** | **20** | **The doctors and other health professionals sometimes ignore what I tell them.** | Item understood and found relevant. 1 patient did not like the word "ignore"; he preferred the alternative "*Doctors and other health professionals don't listen to what I have to say*", but wanted to remove the term "health care professionals". | Reworded  to be less strong and better accepted by the medical community | **U** | **20** | **Many doctors don't listen to what patients tell them.** | Item understood and found relevant by 7/8 patients. | Reworded  to be more categorical | **U** | **20** | **Too many doctors don’t listen to what patients tell them.** |
| **U** | **21** | **My [health problem] has led to financial problems.** | Item understood ("*very clear*") and found relevant. | No change | **U** | **21** | **My [health problem] has led to financial problems.** | Item understood and found relevant. 1 patient thought it was repetitive with item #18. | No change | **U** | **21** | **My [health problem] has led to financial problems.** |
| **U** | **22** | **I find it easy to organize my treatment plan.** | Item not understood by 3/7 patients who suggested to remove it because it was repetitive with item #19. Moreover, the word "organize" was confusing. | Deleted |  |  |  |  |  |  |  |  |
| **U** | **23** | **I find it easy to follow my treatment plan when I am not at home.** | Patients understood this "*good question to ask*". | No change | **U** | **22** | **I find it easy to follow my treatment plan when I am not at home.** | Item understood.  2/8 patients thought it was irrelevant, "*depends on the type of treatment*". One of them found it was repetitive with item #19. | No change | **U** | **22** | **I find it easy to follow my treatment plan when I am not at home.** |
| **U** | **24** | **I am satisfied with the level of information I have about my treatment.** | Item understood and found relevant: "*very good question*". | No change | **U** | **23** | **I am satisfied with the level of information I have about my treatment.** | Item understood and found relevant. | No change | **U** | **23** | **I am satisfied with the level of information I have about my treatment.** |
| **U** | **25** | **I find it easy to manage the different medications I take.** | Item understood by 5/7 patients. | No change | **U** | **24** | **I find it easy to manage the different medications I take.** | Item understood ("*easy question*") and found relevant. | No change | **U** | **24** | **I find it easy to manage the different medications I take.** |
| **U** | **26** | **I find it easy to take my medication for my [health problem].** | Item understood and found similar to item #25. | No change | **U** | **25** | **I find it easy to take my medication for my [health problem].** | Item understood. 4/8 patients thought it was similar to item #24 and suggested to combine items #24 and #25 or to delete item #25. According to 1 patient, *"#24 and #25 are similar but different enough to be appropriate to keep both*". | No change | **U** | **25** | **I find it easy to take my medication for my [health problem].** |
| **R** | **27** | **I am worried about the side effects of some treatments.** | Item understood and found relevant; 1 patient had trouble thinking about this question in the general sense and wanted to associate this with a specific drug/medicine. | Reworded | **R** | **26** | **I am worried about the side effects of some medications.** | Item understood and found relevant, even "*very relevant because every drug has side effects*". 1 patient with breast cancer suggested to add "*at the present time*" because she thinks that this item is "*more relevant when a patient receives chemotherapy*". | No change | **R** | **26** | **I am worried about the side effects of some medications.** |
| **R** | **28** | **Medications should only be taken when needed.** | Item understood but not relevant for 4/7 patients; 2/7 patients suggested to remove it. | Reworded to keep this belief about adherence | **R** | **27** | **I believe I can stop taking my treatment for my [health problem] when I feel better.** | Item understood and found relevant.  1 patient suggested to remove "taking". | Reworded | **R** | **27** | **I believe I can stop my treatment for my [health problem] when I feel better.** |
| **R** | **29** | **I have no control over my [health problem].** | Item understood, but patients thought positive connotation should be used.  This item was found repetitive item #41. It was decided to keep item #41 which has a positive connotation, and to delete this item. | Deleted |  |  |  |  |  |  |  |  |
| **R** | **30** | **I am concerned about becoming too dependent on my treatment.** | Item understood but 4/7 patients did not like it. Some did not like the use of the term "too dependent" and didn't think it applied to their condition. 3 patients wanted to remove this question and 2 wanted to reword it. | Deleted |  |  |  |  |  |  |  |  |
| **R** | **31** | **I am worried about taking medications.** | Item understood, but 2 patients thought items #30 and #31 were similar and wanted to remove #30. | No change | **R** | **28** | **I am worried about taking medications.** | Item understood and mostly found relevant ("Definitely important topic") although 5/8 patients thought it was similar to item #26. Another patient though it was "similar to #26 but different enough to keep both". | No change | **R** | **28** | **I am worried about taking medications.** |
| **R** | **32** | **My [health problem] is very severe.** | Item understood but the use of "very severe" was not appropriate, qualified as "*too much*" or "*jarring*".  "*Serious*" was suggested as an alternative. | Reworded | **R** | **29** | **My [health problem] should be taken seriously.** | Item understood and found relevant. | No change | **R** | **29** | **My [health problem] should be taken seriously.** |
| **R** | **33** | **My [health problem] does not affect my ability to exercise.** | Item understood and found relevant. | No change | **R** | **30** | **My [health problem] does not affect my ability to exercise.** | Item understood and found relevant. 6/8 patients suggested to turn it positive. | Reworded | **R** | **30** | **I am able to exercise despite my [health problem].** |
| **R** | **34** | **My treatment affects my sex life.** | Item understood and found relevant | No change | **R** | **31** | **My treatment affects my sex life.** | Item understood and found relevant. | No change | **R** | **31** | **My treatment affects my sex life.** |
| **R** | **35** | **I am satisfied with the level of information I have about my [health problem].** | Item understood and found relevant: "easy and straight… to the point" | No change | **R** | **32** | **I am satisfied with the level of information I have about my [health problem].** | Item understood and found relevant, "straightforward".  The alternative "*I completely understand my [health problem]*" was preferred by 1 patient; among the 7 other patients, 6 did not have preference. | No change | **R** | **32** | **I am satisfied with the level of information I have about my [health problem].** |
|  |  |  |  |  |  |  |  |  | Alternative added | **R** | **33** | **I completely understand my [health problem].** |
| **R** | **36** | **I don't like taking medications.** | Item understood and found relevant. 1 patient suggested to reword as "*I don't like taking medications, but I know that in my situation they are absolutely necessary*". | No change | **R** | **33** | **I don't like taking medications.** | Item understood and mostly found relevant. 3 patients suggested to remove this item: "*who does?*", *" I don’t think anyone likes taking medications*", "*Doesn't matter*". | No change | **R** | **34** | **I don't like taking medications.** |
| **R** | **37** | **Medications are not good for me.** | Item understood, but 3/7 patients found it was redundant or not relevant and asked to remove it.  2 patients preferred the alternative “*Medications don't do anything for me*”. | Split | **R** | **34** | **The less medication the better.** | Item understood. 4/8 patients thought it was similar to item #33, with "*no value in question after #33*". | Deleted |  |  |  |
|  |  |  |  |  | **R** | **35** | **Medications for my [health problem] don't do anything for me.** | Item understood and mostly found relevant ("*Important question*"). 4/8 patients thought that item #35 was "*better*", "*more specific*" than item #34. | No change | **R** | **35** | **Medications for my [health problem] don't do anything for me.** |
| **R** | **38** | **My treatment helps my [health problem] to improve.** | Item understood and found relevant, but rewording was asked. "Improve" was disturbing for breast cancer, and 1 patient described the confusion as *"breast cancer to improve" meaning "breast cancer to grow"*. | Reworded | **R** | **36** | **My treatment helps my [health problem].** | Item understood and mostly found relevant. 1 patient found this question was "*weird*" and "*did not provide much information*". 2 patients found that items #35 and #36 were similar, and 1 patient thought that "*Question order [was] good for verifying people's answers*". | No change | **R** | **36** | **My treatment helps my [health problem].** |
| **R** | **39** | **There is no point in taking medications for my [health problem].** | Item understood and found relevant. | No change | **R** | **37** | **There is no point in taking medications for my [health problem].** | Item understood and mostly found relevant. 4/8 patients thought it was similar to item #35 but one of them underlined that it was "*more specific*" than item #35. | No change | **R** | **37** | **There is no point in taking medications for my [health problem].** |
| **R** | **40** | **My [health problem] treatment will keep me healthy.** | Item understood but several patients suggested rewording it, e.g., "*My multiple sclerosis treatment will keep me as healthy as I can be*", "*My multiple sclerosis treatment will slow down the progression of multiple sclerosis*", “*My breast cancer treatment will help keep me healthy*”. | Reworded | **R** | **38** | **My [health problem] treatment will keep me as healthy as I can be.** | Item understood by 6/8 patients. 1 patient found it was "*confusing*" and another one reported that "*as healthy as I can be*" did not apply so much. 2 patients suggested to remove this item because they found it "*redundant*" or "*pointless*". | Deleted |  |  |  |
| **R** | **41** | **My actions directly affect my [health problem].** | Item understood but 2 patients did not like the wording, and 1 patient wanted to clarify the word "actions". | Reworded | **R** | **39** | **What I do impacts on my [health problem].** | Comprehension of this question was heterogenous. "What I do” was "*vague/unclear*" for 1 patient, and another wondered "*Other than the medications, what I do*?" 4/8 patients agreed with the wording, once the typo/grammar ("on") was removed. | Reworded | **R** | **38** | **What I do impacts my [health problem].** |
| **R** | **42** | **My [health problem] is unlikely to get worse whether I follow my treatment plan or not.** | Item understood but 1 patient was confused by the use of the term "unlikely", and 2 patients suggested to reword as: "*My diabetes [or multiple sclerosis] is likely to get worse if I don't follow my treatment plan*". | Reworded | **R** | **40** | **My [health problem] is likely to get worse if I don't follow my treatment plan.** | Item understood and mostly found relevant. 1 patient found this item obvious ("*if you don't follow your treatment, of course it's going to get worse*") and another found it repetitive. | No change | **R** | **39** | **My [health problem] is likely to get worse if I don’t follow my treatment plan.** |
| **R** | **43** | **I feel worse if I don't follow my treatment plan.** | Item understood and found relevant.  1 patient suggested to replace "worse" by "guilty", but this suggestion seemed to only apply to her. | No change | **R** | **41** | **I feel worse if I don't follow my treatment plan.** | Item understood. The patient who found item #40 obvious also found this item obvious ("*of course you are going to get worse*"). This patient and 2 other patients found this item redundant with item #40 and suggested to remove it. | No change | **R** | **40** | **I feel worse if I don't follow my treatment plan.** |
| **U** | **44** | **Medications are more expensive than they should be.** | Item understood by all patients and found relevant ("*great question*") by 6/7 patients. | No change | **U** | **42** | **Medications are more expensive than they should be.** | Item understood and found relevant, although the answer seemed obvious for 3 patients: "*everyone will answer "5"*", “*treatment is required for breast cancer whether expensive or not*". | No change | **U** | **41** | **Medications are more expensive than they should be.** |
| **P** | **45** | **My [health problem] keeps me from doing things I want to do.** | Item understood and found relevant. | No change | **P** | **43** | **My [health problem] keeps me from doing things I want to do.** | Item understood by 7/8 patients and found relevant by 6/8 patients. 2 patients suggested to remove this item because they found it redundant: "*topic/concept covered earlier*". | No change | **P** | **42** | **My [health problem] keeps me from doing things I want to do.** |
| **P** | **46** | **Following my [health problem] treatment plan lets me do the things I want to do.** | Item understood by 6/7 patients; 1 patient thought it meant "*If you do listen to your doctor, you will be able to do everything you want*." | No change | **P** | **44** | **Following my [health problem] treatment plan lets me do the things I want to do.** | Item understood and found repetitive by 5/8 patients. The 3 other patients found this item "*useful*", "*to the point*", and 2 of them underlined that it “*flowed well*” after item #43. | No change | **P** | **43** | **Following my [health problem] treatment plan lets me do the things I want to do.** |
| **R** | **47** | **I focus better when I am on my treatment plan.** | Item understood and found relevant by all patients. | No change | **R** | **45** | **I focus better when I am on my treatment plan.** | Item understood and found relevant, except for 3 patients: 1 patient found it repetitive, another patient found it not relevant because his treatment did not work, and another patient found it “*inappropriate for breast cancer*”. | Deleted |  |  |  |
| **R** | **48** | **I believe non-traditional treatments can replace some of my medications.** | Item understood by 6/7 patients; 1 patient was struggled with it. 1 patient suggested to replace "can" by "could". | Reworded | **R** | **46** | **Non-traditional treatments could replace some of my medications.** | Item understood and found relevant by all except 1 patient who "*doesn't know anything about "non-traditional" treatment*". | No change | **R** | **44** | **Non-traditional treatments could replace some of my medications.** |
| **P** | **49** | **I have found ways to deal with my [health problem].** | Item understood but found repetitive by 1 patient. | No change | **P** | **47** | **I have found ways to deal with my [health problem].** | Item understood and found relevant. | No change | **P** | **45** | **I have found ways to deal with my [health problem].** |

**Abbreviations: P**, Psychological**;** **R**, Rational; **S**, Social; **SPUR**, Social, Psychological, Usage and Rational; **U**, Usage
