# Supplementary material for: Development of the SPUR tool: a profiling instrument for patient treatment behavior
Source: J Patient Rep Outcomes. 2022 Jun 6;6:61. doi: 10.1186/s41687-022-00470-x (PMC9170867; doi:10.1186/s41687-022-00470-x)
Supplement: Supplementary file 4 — Additional file 4: SPUR Tool Pilot Version. [file 41687_2022_470_MOESM4_ESM.docx]

# Supplementary material: SPUR Tool Pilot Version

The patient is instructed to select a number between 1 to 5 that best applied to them, with 1=“I strongly disagree” and 5=“I strongly agree”.

| S | 1. My [health problem] affects my relationships with those I care about. |
| --- | --- |
| S | 1. My [health problem] affects my social life. |
| S | 1. I would be interested in knowing if others with [health problem] follow their treatment plan. |
| S | 1. I think that people with [health problem] generally follow their doctors' prescription exactly. |
| S | 1. The people in my life help me manage my [health problem]. |
| P | 1. Fighting for my health is my highest priority. |
| P | 1. Precisely following doctors’ recommendations is the best way for me to stay healthy. |
| P | 1. I trust doctors' recommendations. |
| P | 1. It is essential that I follow my treatment plan. |
| P | 1. Sometimes my [health problem] seems unreal to me. |
| P | 1. I'm the kind of person who will follow their treatment plan exactly. |
| P | 1. I will have to take a treatment for my [health problem] for the rest of my life. |
| P | 1. I live in the moment. |
| P | 1. If my doctor recommends that I do something, I do it. |
| P | 1. Sometimes doctors prescribe treatment you don't really need. |
| P | 1. Sometimes I don't follow my treatment plan exactly. |
| U | 1. I find it easy to get my treatment for my [health problem]. |
| U | 1. I can easily pay for my treatment. |
| U | 1. I am able to follow my treatment plan. |
| U | 1. Too many doctors don’t listen to what patients tell them. |
| U | 1. My [health problem] has led to financial problems. |
| U | 1. I find it easy to follow my treatment plan when I am not at home. |
| U | 1. I am satisfied with the level of information I have about my treatment. |
| U | 1. I find it easy to manage the different medications I take. |
| U | 1. I find it easy to take my medication for my [health problem]. |
| R | 1. I am worried about the side effects of some medications. |
| R | 1. I believe I can stop my treatment for my [health problem] when I feel better. |
| R | 1. I am worried about taking medications. |
| R | 1. My [health problem] should be taken seriously. |
| R | 1. I am able to exercise despite my [health problem]. |
| R | 1. My treatment affects my sex life. |
| R | 1. I am satisfied with the level of information I have about my [health problem]. |
| R | 1. I completely understand my [health problem]. |
| R | 1. I don't like taking medications. |
| R | 1. Medications for my [health problem] don't do anything for me. |
| R | 1. My treatment helps my [health problem]. |
| R | 1. There is no point in taking medications for my [health problem]. |
| R | 1. What I do impacts my [health problem]. |
| R | 1. My [health problem] is likely to get worse if I don’t follow my treatment plan. |
| R | 1. I feel worse if I don't follow my treatment plan. |
| U | 1. Medications are more expensive than they should be. |
| P | 1. My [health problem] keeps me from doing things I want to do. |
| P | 1. Following my [health problem] treatment plan lets me do the things I want to do. |
| R | 1. Non-traditional treatments could replace some of my medications. |
| P | 1. I have found ways to deal with my [health problem]. |

P: Psychological; U: Usage; R: Rationale; S: Social
